# Medicinal plants and compounds for chronic bronchitis treatment: efficacy and action mechanisms

**DOI:** 10.3389/fphar.2025.1674079

**Published:** 2025-10-29

**Authors:** Wei Ding, Danni Chen, Jiawang Li, Yan Wang, Xiangyun Chen, Yihe Xu, Zhen Yang, Zhenhong Liu, Hongxia Zhao

**Affiliations:** 1 College of Traditional Chinese Medicine, Beijing University of Chinese Medicine, Beijing, China; 2 Chinese PLA General Hospital, Interventional Ultrasound Department, Beijing, China; 3 Institute for Brain Disorders, Dongzhimen Hospital, Beijing University of Chinese Medicine, Beijing, China; 4 Institute of Basic Theory for Chinese Medicine, China Academy of Chinese Medical Sciences, Beijing, China

**Keywords:** chronic bronchitis (CB), chronic obstructive pulmonary disease (COPD), phytotherapy, natural compounds, medicinal plants

## Abstract

**Background:**

Chronic bronchitis (CB) is a common yet heterogeneous condition characterized by persistent inflammation, oxidative stress, airway hyperresponsiveness, and mucus hypersecretion. As an early stage of various severe pulmonary diseases, current therapeutic strategies remain unsatisfactory. Substantial evidence indicates that medicinal plants and compounds hold potential for treating inflammatory lung disorders. This study aims to consolidate recent and reliable evidence concerning the multi-targeted roles and underlying molecular mechanisms of these natural products in the treatment of CB.

**Methods:**

This systematic review followed a prospectively registered protocol (PROSPERO ID: CRD42024588912). A comprehensive literature search encompassed multiple electronic databases, including PubMed, Scopus, Embase, Web of Science, VIP, Wan-fang, SinoMed, and the China National Knowledge Infrastructure Study selection strictly adhered to the PICOS principles to systematically identify medicinal plants and compounds with therapeutic potential against Chronic bronchitis.

**Results:**

The results identified 13 medicinal plants and 19 compounds that exhibited anti-inflammatory activity. Additionally, 8 plants and 12 compounds demonstrated further therapeutic effects, including antioxidant, anti-mucus, and potential bronchodilatory activities. The underlying mechanisms primarily involved the NF-κB, PI3K/AKT/mTOR, TLR4, MAPK, and Nrf2 pathways. Ursolic acid emerged as the most promising clinical candidate.

**Conclusion:**

This review represents the first comprehensive synthesis of experimentally verified efficacy and mechanisms associated with medicinal plants and compounds in CB treatment. Preclinical animal studies have confirmed the therapeutic benefits of these natural agents in alleviating CB symptoms, establishing a solid foundation for novel drug development and underscoring their considerable translational potential.

## 1 Introduction

**FIGURE 1:**
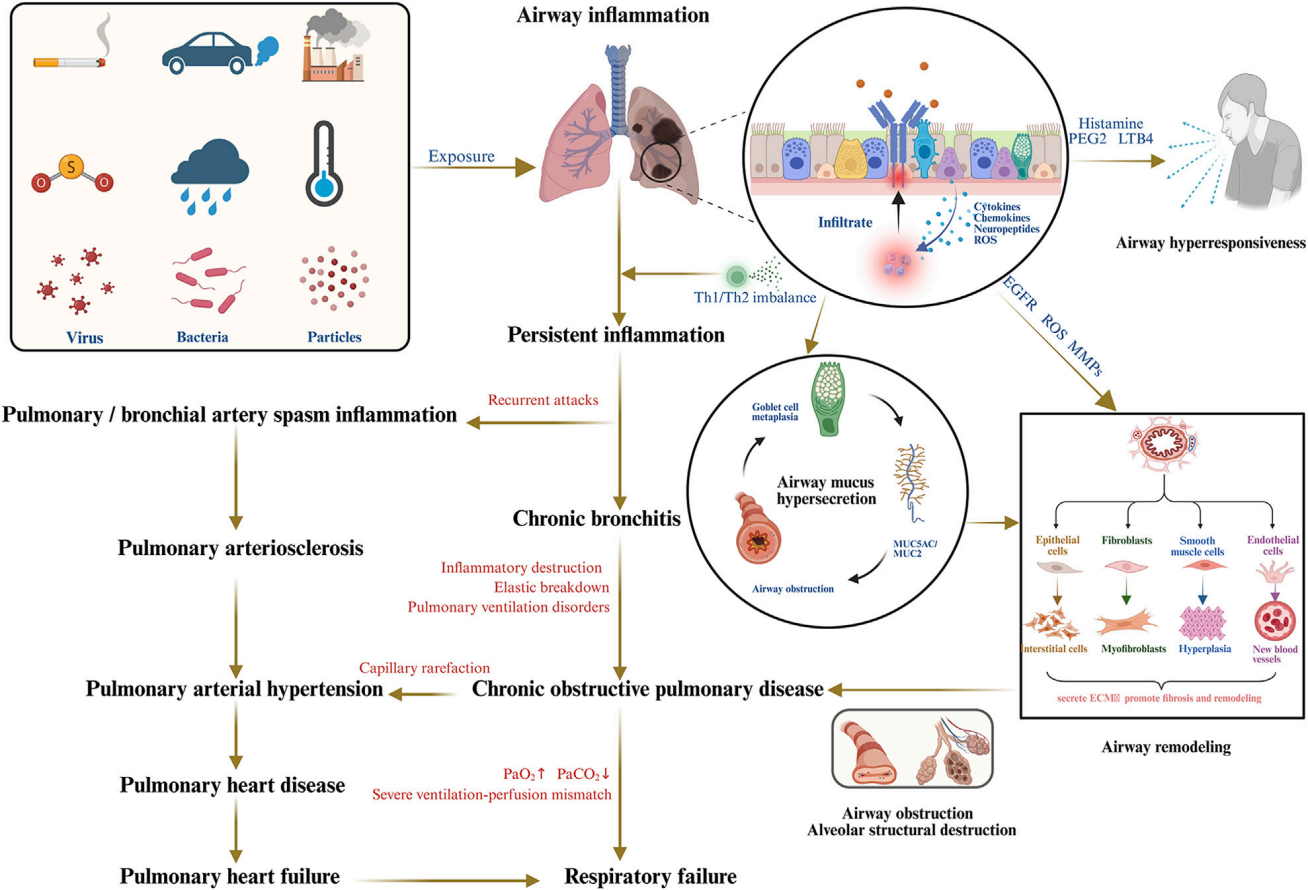
The pathogenesis of CB and its serious complications. [Created in Bio Render Ding, W. (2025), Agreement number: KI28UULM29].

Chronic bronchitis (CB) is a common and treatment-resistant progressive respiratory disorder characterized by persistent tracheobronchial inflammation and frequently leading to serious complications. As the predominant clinical phenotype of chronic obstructive pulmonary disease (COPD), CB represents a significant risk factor for respiratory-related mortality and contributes substantially to the global disease burden (Guo et al., 2006; Pelkonen et al., 2006). With COPD affecting 384 million people and causing over 3.23 million deaths annually, CB is estimated to account for 14%–74% of this burden (GBD, 2019 Diseases and Injuries Collaborators, 2020; Christenson et al., 2022; Kim and Criner, 2013). However, COPD diagnosis rates remain critically low at below 30%, resulting in numerous undetected cases and fueling a major public health crisis often referred to as the “hidden epidemic” (Fu et al., 2025). Accordingly, elucidating the underlying pathological mechanisms is essential for guiding therapeutic strategies and improving prognostic outcomes in CB (Al-Jahdali et al., 2025). Its pathogenesis arises from a dynamic interplay of recurrent and persistent inflammatory processes, oxidative stress, airway hyperresponsiveness, mucus hypersecretion, and airway remodeling (Figure 1). Despite advances in understanding CB pathogenesis in recent years, no breakthrough therapies have emerged. Current controller treatments, including bronchodilators, inhaled corticosteroids, and mucolytic agents, have not been shown to significantly reverse lung function decline, reduce mortality, or sustainably improve patient outcomes (M et al., 2020).

Medicinal plants and their derivatives hold significant importance in the medical field and have been utilized throughout human history. Over recent decades, reliance on these plants as a cost-effective and safe form of complementary and alternative medicine has grown steadily (Frishman et al., 2009). Medicinal plants form the cornerstone of major traditional medical systems such as Chinese, Ayurveda, and Unani Tibb, which are practiced globally and serve as the primary therapeutic choice for over 80% of the world’s population (Al Disi et al., 2016). Currently, approximately 70% of people in developed countries use medicinal plants, and about 25% of prescribed drugs in the United States contain at least one botanical component (Rajizadeh et al., 2024). Their impact is even greater in developing nations (WHO, 2003). Numerous phytochemicals, including morphine, artemisinin, ephedrine, and paclitaxel, have been scientifically validated for their notable bioactivities (Chen et al., 2025). Remarkably, plant-derived compounds form the chemical foundation of over 50% of all approved pharmaceuticals currently in use (Pan et al., 2013). Recent studies have demonstrated considerable progress in applying natural products to manage pulmonary diseases, attributable to their anti-inflammatory, antioxidant, antimicrobial, and antiviral properties (Rahman et al., 2022). These properties enable multi-targeted interventions against the pathological processes of CB, making them a promising therapeutic approach.

While natural products are highly regarded for their therapeutic potential, no systematic review has specifically addressed their application in treating CB. To address this gap, this review systematically synthesizes the existing literature on natural products, clarifies their mechanisms of action, and evaluates their potential for clinical management of CB.

## 2 Methods

This review was prospectively registered with PROSPERO (CRD42024588912) and conducted in accordance with the PRISMA guidelines.

### 2.1 Literature identification

The literature search, covering records from inception to 30 August 2024, was performed across eight databases: PubMed, Scopus, Web of Science, Embase, China National Knowledge Infrastructure (CNKI), SinoMed, Wan-fang, and VIP. The search strategy employed a combination of Medical Subject Headings (MeSH) terms and free-text phrases, such as “CB,” “plants,” “herbs,” and “phytochemicals,” and was restricted to English and Chinese publications. The detailed search strategy for each database is provided in the Supplementary Material.

### 2.2 Eligibility criteria

**FIGURE 2:**
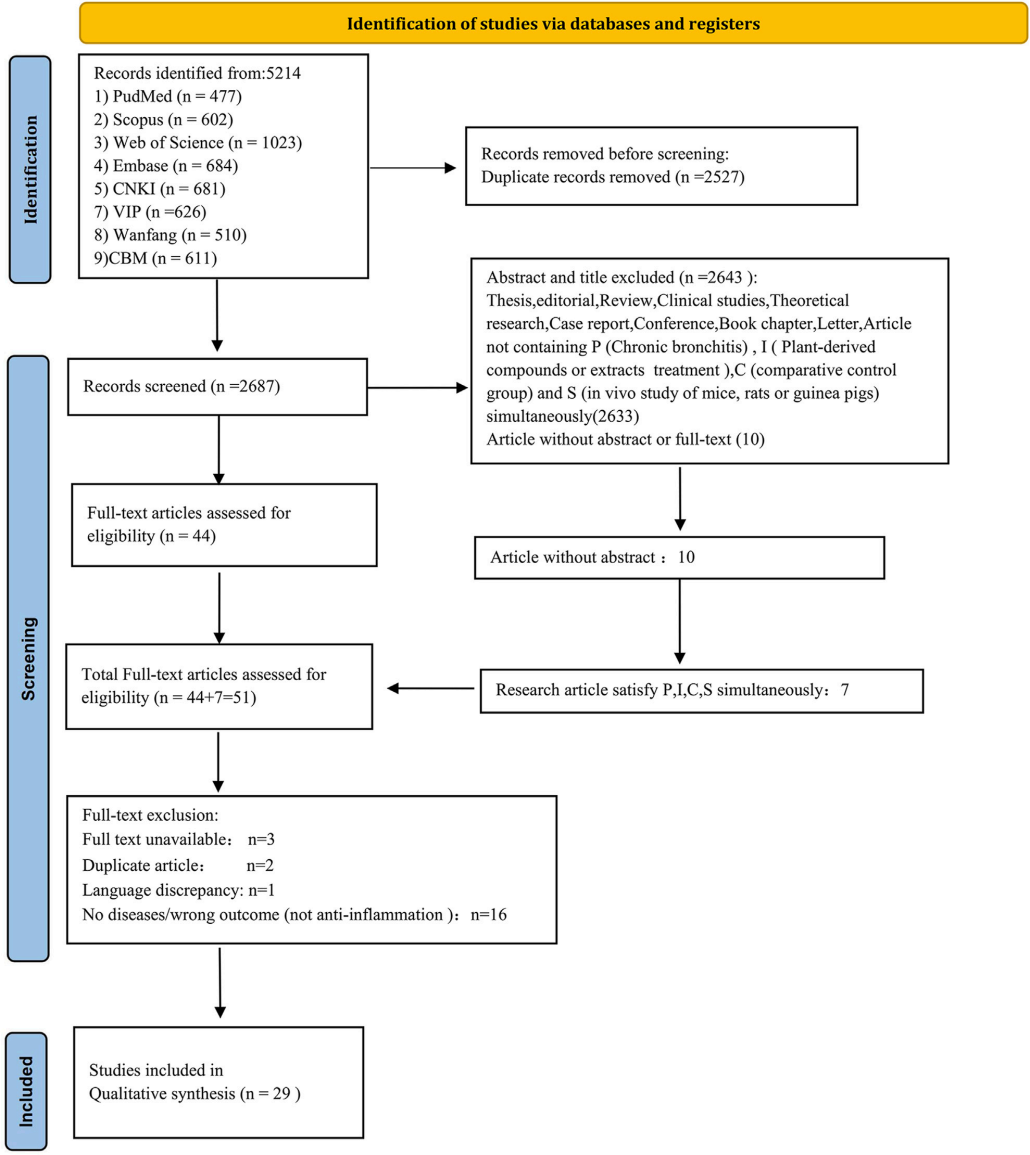
PRISMA flowchart illustrating the process of identifying eligible studies.

The PICOS principle served as the gold standard for defining inclusion and exclusion criteria. Inclusion criteria were defined as follows: P (Patients): CB; I (Intervention): medicinal plant extracts and plant-derived compounds; C (Comparison): comparative control group; O (Outcome): anti-inflammatory potency; and S (Study type): *in vivo* studies involving rats, mice, or guinea pigs. Exclusion criteria were as follows: theses, editorials, reviews, clinical studies, theoretical research, case reports, conferences, book chapters, letters, unpublished grey literature, and articles not satisfying PICOS conditions. After eliminating duplicate entries, two independent reviewers assessed the literature based on titles and abstracts. Studies meeting the predetermined criteria were selected for further review, and full texts were retrieved and evaluated for eligibility according to predefined inclusion and exclusion criteria. Figure 2 illustrates the article selection process.

### 2.3 Data extraction

Data extracted from the 29 included studies encompassed research characteristics (first author’s name, publication year), animal species studied, models utilized to induce CB, medicinal plant information (plant source, family, extraction solvent, plant part extracted), and primary outcome measures related to inflammatory responses (lung or airway histological injury evidence, inflammatory markers, and edema conditions). Secondary outcomes included oxidative stress, airway hyperresponsiveness (AHR), airway mucus hypersecretion (AMH), and other relevant measures. These extracted data are summarized in Tables 1–7.

**TABLE 1:** Medicinal plants in the treatment of CB.

Medicinal plants (Family)	Extract/part	Dose/duration	Species/model	Inflammation	Oxidation	AHR	AMH	Refs
*Aconitum septentrionale* Koelle (Ranunculaceae)	Hydroalcoholic extract/body	0.3, 0.6, 0.9 mg/kg, 20 days	Wistar rats, LPS (0.2 mg/mL/days, 7 days)	Lung histopathological changes↓ Lung IL-1β↓				Han et al. (2011)
*Ajuga decumbens * Thunb. (Lbiatae)	Aqueous extract/leaf	100, 200, 400 mg/kg, 15 days	KM mice, Cigarette smoke (5 cigarettes/30 min, 2/days, 26 days) + LPS (1 mg/mL, twice)	Lung histopathological changes↓ BALF leukocytes and neutrophils↓ Plasma MPO activity↓	Plasma SOD, GSH-Px↑ MDA↓			Xiong et al. (2016)
*Atalantia buxifolia* (Poir)*.* Oliv. ex Benth (Rutaceae)	Hydroalcoholic extract/root	2.5, 5, 10 g/kg, 10 days	SD rats, SO_2_ smoke (3 g/30 min/, qd, 28 days)	Lung histopathological changes↓ TNF-α↓, IL-4, IL-10↑ in Lung tissue homogenate				Yin et al. (2014)
*Bambusae caulis in taeniam* (Poaceae)	Aqueous extract/stems	50,100 mg/kg, 28 days	C57BL/6 mice, Cigarette smoke (5 cigarettes, 30 min/days, 5 days/wk, 4weeks)	Lung histopathological changes↓ BALF IL-1β, IL-6, TNF-α, CCL2↓ BALF total cells, neutrophils, macrophages, lymphocytes↓				Kim et al. (2018)
*Citrus grandis* (L.) Osbeck (Rutaceae)	Ethanolic extract/epicarp	247, 493, 986 mg/kg, 7 days	NIH mice, Ammonia liquor (0.2 mL); Xylene (0.05 mL)	Ear swelling↓		Cough↓ Latent period of cough↑	Expectorant activity↑	Jiang et al. (2014)
*Elaeagnus pungens* Thunb. (Elaeagnaceae)	Aqueous extract/leaf	2.5, 5 g/kg, 5 days	KM mice, Ammonia liquor (constant Pressure); Xylene (15 mL)	Ear swelling↓		Cough↓ Latent period of cough↑		Zhu et al. (2018)
*Eucalyptu globulus* Labill. (Myrtaceae)	Myrtaceae extract/leaf	30,100,300 mg/kg, 21 days	SD rats, LPS (1 mg/mL, once)	Lung histopathological changes↓ Total cells, neutrophils, lymphocytes, macrophages in BALF↓			BALF MUC5AC↓ MUC5AC protein expression↓	Lu et al. (2004)
*Gentiana veitchiorum* Hemsl. (Gentianaceae)	Hydroalcoholic extract/body	4 g/kg, 15 days	KM mice, Ammonia spray inhalation (2 min, 5/days, 30 days)	Lung histopathological changes↓ Serum IL-10↑, TNF-α↓	Lung SOD, T-AOC activity↑			Geng et al. (2010)
*Glycyrrhiza uralensis* Fisch*.* ex DC. (Fabaceae)	Aqueous extract/root	10 g/kg, bid, 60 days	KM mice, Cigarette smoke (30 min, 4/days, 60 days)	Lung histopathological changes↓	Lung SOD↑, MDA↓			Chen et al. (2007)
*Inula japonica* Thunb. (Asteraceae)	Hydroalcoholic extract/flower	0.25, 0.5 and 1 g/kg, 56 days	Wistar rats Cigarette smoke (2 cigarettes/30 min, 2/days, 4 weeks and 2 cigarettes/30 min, 2/days, 4 weeks) + LPS (200 μg/μL, twice)	Lung histopathological changes↓ Inflammatory cells in BALF↓ Serum IL-6, IL-17, PGE2, COX-2, AP-1↓ IL-10↑	Serum NO, iNOS↓	PIF, PEF, TV, F, FEF50↑, Ti, Te, RT, Pau↓		Ma (2023)
*Lysionotus pauciflorus* Maxim. (Gesneriaceae)	Aqueous extract/body	2, 8 g/kg, 28 days	Wistar rats, Cigarette smoke (4 cigarettes/30 min, 2/days, 26 days) +LPS (200 μg/μL, 0.2 mL, twice)	Lung histopathological changes↓ TLR4 mRNA↓				Zhao et al. (2018)
*Phyllanthus emblica *L. (Phyllanthaceae)	Ethyl acetate extract/root	50,100,150 mg/kg, 14 days	Wistar rats, Cigarette smoke (20 cigarettes/1 h, 2/days, 33 days) + LPS (0.2 mL, twice)	Lung histopathological changes↓ BALF total cells, neutrophils, lymphocytes, macrophages↓				Zhong and Zeng (2008)
*Rohdea fargesii* var. *Fargesii* (Asparagaceae)	Hydroalcoholic extract/body	100,500 mg/kg, 5 days	KM mice, ammonia liquor (25%–28%,0.1 mL); Xylene (0.02 mL per mouse); Cigarette smoke (1cigarettes/60 min, 2/days, 20 days and 1 cigarettes/60 min, 1/days, 20 days)	Lung histopathological changes↓ Serum IL-6, TNF-α↓ Ear swelling↓		Cough↓ Latent period of cough↑	Expectorant activity↑	Li (2008)

**TABLE 2:** Plant-derived flavonoids in the treatment of CB.

Compounds	Classification	Plant source	Dose/duration	Species/model	Inflammation	Oxidation	AHR	AMH	Refs
Flos Inulae flavonoid	flavonoids	*Inula japonica* Thunb. (Asteraceae)	0.005 g/kg, 56 days	Wistar rats Cigarette smoke (2 cigarettes/30 min, 2/days, 4 weeks and 2 cigarettes/30 min, 2/days, 4 weeks) + LPS (200 μg/μL, twice)	Lung histopathological changes↓ IL-6, COX-2, AP-1 in serum↓, IL-10 in serum↑	Serum iNOS↓			Ma (2023)
Kaempferol	flavonoids	*Elaeagnus pungens *Thunb. (Elaeagnaceae)	50,100 mg/kg, 5 days	KM mice, Ammonia liquor (constant Pressure); Xylene (15 mL)	Ear swelling↓		Cough↓ Latent period of cough↑		(Zhu et al., 2018)
Naringin	flavonoids	*Citrus grandis* (L.) Osbeck (Rutaceae)	9.2,18.4,36.8 mg/kg, 56 days	Hartley Guinea pig, Cigarette smoke (10 cigarettes/1 h, 1/days, 6 days/weeks, 8 weeks)	Lung histopathological changes↓ BALF IL-8, TNF-α, LTB4↓, LXA4↑ Total cells, leukocytes, neutrophils, lymphocytes, monocytes, eosinophils, basophils in BALF↓ BALF and lung MPO activity ↓	Lung SOD activity ↑	Cough↓ Latent period of cough↑		Luo et al. (2012)
Quercetin	flavonoids	*Elaeagnus pungens* Thunb. (Elaeagnaceae)	50,100 mg/kg, 5 days	KM mice, Ammonia liquor (constant Pressure); Xylene (15 mL)	Ear swelling↓		Cough↓ Latent period of cough↑		Zhu et al. (2018)
Quercetin	flavonoids	*—*	25, 50 mg/kg, 28 days	SD rats Cigarette smoke (5 cigarettes/30 min, 2/days, 6 days/weeks, 4 weeks)	Lung histopathological changes↓ Total cells, leukocytes, neutrophils, lymphocytes, monocytes in BALF↓ BALF IL-8, TNF-α↓ Lung p-P65 protein expression↓ IκBα and p-EGFR protein expression↑	Lung GSH, T-AOC↑		Intracellular mucous glycoconjugates↓ MUC5AC mRNA and protein expression↓	Yang et al. (2012)
Scutellarin	flavonoids	*Scutellaria baicalensis* Georgi (Lamiaceae)	40 mg/kg, 13 days	SD rats Cigarette smoke (10 cigarettes/30 min, 2/days, 58 days) + LPS (1.5 mg/mL, twice)	Lung histopathological changes↓ BALF protein content, leukocytes and neutrophils↓ Serum TNF-α, IL-17A↓, IL-10↑ BALF IL-6, IL-1β↓ PI3K, AKT, and mTOR mRNA↓ p-PI3K, p-AKT, and p-mTOR protein expression↓	Lung SOD↑, MDA↓			Wang et al. (2023a)
Seabuckthorn flavone	flavonoids	*Hippophae rhamnoides* L. (Elaeagnaceae)	100,200,500 mg/kg, 30 days	KM mice Cigarette smoke (12 cigarettes/20 min, 2/days, 26 days) +LPS (1 mg/mL, twice)	Lung histopathological changes↓ leukocytes, neutrophils, macrophages in BALF ↓ IL-1β, IL-6, COX-2, CXCL1 protein expression↓			MUC5AC protein expression↓	Ren (2019)

**TABLE 3:** Plant-derived polyphenols in the treatment of CB.

Plant-derived compounds	Classification	Plant source	Dose/duration	Species/model	Inflammation	Oxidation	AHR	AMH	Refs
Bergenin	Polyphenols	*Bergenia purpurascens* (Hook.f. and Thomson) Engl. (Saxifragaceae)	87.5 mg/kg, 28 days	SD rats Cigarette smoke (60% tobacco/1 h, 1/days, 28 days)	Lung histopathological changes↓ Leukocytes, neutrophils, macrophages, lymphocytes in BALF ↓				Zhang et al. (2019)
*P*-coumaric acid	Polyphenols	*Phyllostachys nigra* var*. henonis (*Mitford) Rendle (Poaceae)	5,10 mg/kg, 28 days	C57BL/6 mice, Cigarette smoke (5 cigarettes, 30 min/days, 5 days/weeks, 4weeks)	Lung histopathological changes↓ IL-1β, IL-6, TNF-α, CCL2 in BALF ↓ Total cells, neutrophils, macrophages, lymphocytes in BALF ↓ Lung tissue and nuclear NF-κB p65 protein expression↓				Kim et al. (2018)
Polydatin	Polyphenols	*Reynoutria japonica* Houtt. (Polygonaceae)	60 mg/kg, 13 days	SD rats Cigarette smoke (10 cigarettes/30 min, 2/days, 58 days) +LPS (1.5 mg/mL, twice)	Lung histopathological changes↓ BALF protein content, leukocytes and Neutrophils↓ Serum TNF-α, IL-17A↓, IL-10↑ BALF IL-6, IL-1β↓ PI3K, AKT, and mTOR mRNA↓ p-PI3K, p-AKT, and p-mTOR protein expression↓	Lung SOD↑, MDA↓			Wang et al. (2023a)
Punicalagin	Polyphenols	*Punica granatum* L. (Lythraceae)	10, 20, 40 mg/kg, 15 days	Sawdust 70 g, tobacco 30 g, pepper 15 g, sulfur 1 g (40 min/days, 20 days) and Sawdust 70 g, tobacco 40 g, pepper 15 g, sulfur 1 g (30 min/days, 20 days)	Serum TNF-α, IL-8, IL-1β, IL-6↓	SOD, GSH-Px, CAT↑, MDA↓ PPARγ, PGC-1α, Nrf2, γ-GCS protein expression↑			Shang et al. (2017)
Mangiferin	Polyphenols	*Mangifera indica* L. (Anacardiaceae)	100, 200 mg/kg, 28 days	SD rats, Cigarette smoke (10 cigarettes/1 h, 2 h/days, 6 weeks)	Lung histopathological changes↓ hs-CRP, TNF-α↓ NF-κBp65, IκBα protein expression↓ NF-κB p65 mRNA, IκBα mRNA↓				Wei et al. (2014)

**TABLE 4:** Plant-derived terpenoids in the treatment of CB.

Plant-derived compounds	Classification	Plant source	Dose/duration	Species/model	Inflammation	Oxidation	AHR	AMH	Refs
Flos Inulae sesquiterpene	Terpenoids	*Inula japonica* Thunb. (Asteraceae)	0.03 g/kg, 56 days	Wistar rats Cigarette smoke (2 cigarettes/30 min, 2/days, 4 weeks and 2 cigarettes/30 min, 2/days, 4 weeks) + LPS (200 μg/μL, twice)	Lung histopathological changes↓ Inflammatory cells in BALF↓ IL-6, IL-17, COX-2 in serum↓, IL-10 in serum↑ NF-κBp65 and IκBα protein expression↓	Serum iNOS↓	Te, RT, Pau, F, EEF↓		Ma (2023)
Glycyrrhizin	Terpenoids	*Glycyrrhiza uralensis* Fisch. ex DC. (Fabaceae)	2.5, 5, 10 mg/kg, 14 days	KM mice Ammonia (2 mL/30 min, 2/days, 30 days) + Cigarette smoke (400 mL/30 min, 2/days, 30 days)	TNF-α, IL-1β↓ TNF-α, IL-1β mRNA↓ TNF-α, IL-1β protein expression↓				Wang and Chen (2016)
Ursolic acid	Terpenoids	*Eriobotrya japonica* (thunb.) Lindl (Rosaceae)	50, 150, 450 mg/kg, 14 days	SD rats, BCG (5 mg/kg) + LPS (200 μg/μL)	Lung histopathological changes↓ Leukocytes, neutrophils and alveolar macrophages in BALF↓ TNF-α, IL-1β, PGE2, LTB4, IL-8 in AM↓ TNF-α, IL-8 in lung homogenate↓ IL-10 in lung homogenate↑ NF-κB p65, ERK, p38, Phospho-p38, TNF-α protein expression↓ TNF-α mRNA↓ ICAM-1 protein expression↓	MDA, HO-1, iNOS activity↓ NO and iNOS concentration↓ SOD activity↑ HO-1 and iNOS mRNA↓ HO-1 and iNOS protein expression↓			Huang et al. (2006), Huang et al. (2009), Huang et al. (2007), Ge et al. (2009), Jiang et al. (2009)

**TABLE 5:** Plant-derived polysaccharides in the treatment of CB.

Plant-derived compounds	Classification	Plant source	Dose/duration	Species/model	Inflammation	Oxidation	AHR	AMH	Refs
Platycodon grandiflorus polysaccharide	Polysaccharides	Platycodon grandiflorus (Jacq.) A. DC. (Campanulaceae)	75, 150, 300 mg/kg, 15 days	Wistar rats, 2% (v/v) SO2 smoke (30 min/days, 15 days)	Lung histopathological changes↓ TNF-α↓ TNF-α protein expression↓ BALF Th1↓, Th2↑, IFN-γ↓, IL-4↑	NO, iNOS in lung↓		Mucin-positive area↓ MUC2 protein expression↓	Liu et al. (2022)
Flos inulae polysaccharide	Polysaccharides	*Inula japonica* Thunb. (Asteraceae)	0.17 g/kg, 56 days	Wistar rats Cigarette smoke (2 cigarettes/30 min, 2/days, 4 weeks and 2 cigarettes/30 min, 2/days, 4weeks) + LPS (200 μg/μL, twice)	Lung histopathological changes↓ IL-6 in serum↓ IL-10 in serum↑				(Ma, 2023)

**TABLE 6:** Plant-derived saponins in the treatment of CB.

Plant-derived compounds	Classification	Plant source	Dose/duration	Species/model	Inflammation	Oxidation	AHR	AMH	Refs
platycodin D	Saponins	*Platycodon grandifloru*s (Jacq.) A. DC. (Campanulaceae)	2 mg/kg, 15 days	Wistar rats, 2% (v/v) SO2 smoke (30 min/days, 15 days)	Lung histopathological changes↓ TNF-α↓ TNF-α protein expression↓ BALF Th1, Th2↓ IFN-γ↓, IL-4↑	NO, iNOS in lung↓		Mucin-positive area↓ MUC2 protein expression↓	Liu et al. (2022)
Kikyosaponin	Saponins	*Platycodon grandiflorus* (Jacq.) A. DC. (Campanulaceae)	12.5, 25, 50 mg/kg, 30 days	KM mice, Ammonia (2 mL/30 min, 2/days, 30 days) + Cigarette smoke (400 mL/30 min, 2/days, 30 days)	Lung histopathological changes↓ TNF-α, IL-1β↓ TNF-α, IL-1β protein expression↓				He et al. (2013)

**TABLE 7:** Plant-derived alkaloids in the treatment of CB.

Plant-derived compounds	Classification	Plant source	Dose/duration	Species/model	Inflammation	Oxidation	AHR	AMH	Refs
Aconitum total alkaloids	Alkaloids	*Aconitum septentrionale* Koelle (Ranunculaceae)	50,100 mg/kg, 14 days	SD rats, BCG (25 mL/kg) + LPS (1 mg/kg)	Lung histopathological changes↓ Serum IL-1, IL-6, TNF-α↓				Hong et al. (2019)

### 2.4 Quality assessment

**FIGURE 3:**
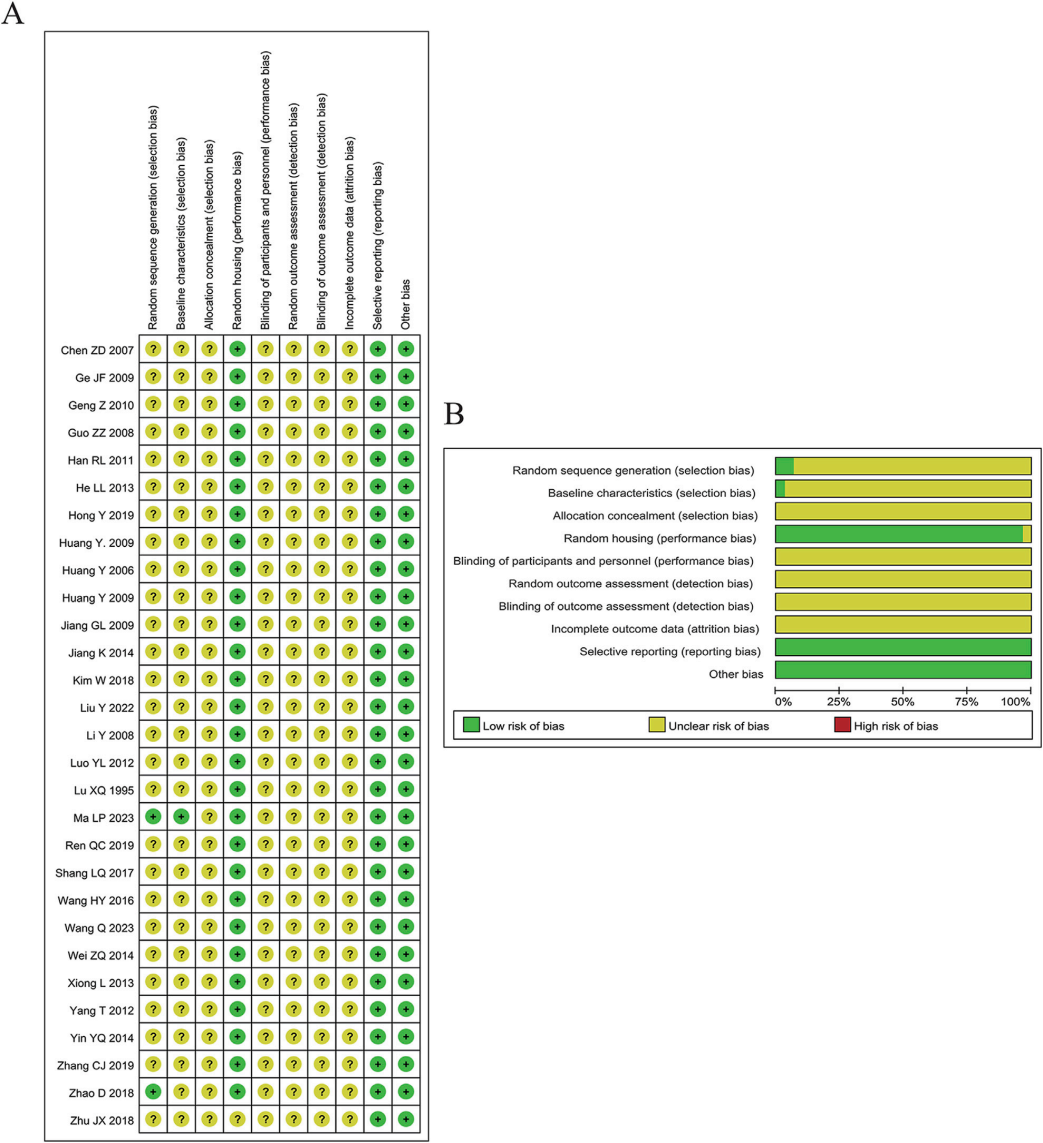
Risk of bias appraisal. **(A)** Risk of bias summary. **(B)** Risk of bias graph.

To evaluate methodological rigor, SYRCLE’s risk of bias tool was employed for the animal studies included in our analysis. This tool examines ten distinct criteria across six domains, assigning each a rating of high risk, low risk, or unclear risk depending on details provided by each study. Wei Ding and Danni Chen independently conducted the quality assessment, resolving disagreements through discussion until consensus was achieved. Figure 3 present the risk of bias in the included studies.

## 3 Results

### 3.1 Study inclusion

From the initial 5,214 articles identified, 2,527 duplicates were removed. Abstract and title screening led to the exclusion of 2,633 additional studies. Among ten articles lacking abstracts, seven satisfied the criteria for P, I, C, and S simultaneously. Of the remaining 51 articles, three could not be retrieved, two were duplicated, and one was in Mongolian. A further 16 studies were excluded due to incorrect outcomes (lack of anti-inflammatory data). Ultimately, 29 investigations, conducted between 1995 and 2024, were included, each featuring *in vivo* trials with rats, mice, or guinea pigs. Figure 2 provides a systematic and comprehensive flow diagram.

### 3.2 Risk of bias assessment

As shown in Figure 3, although these studies were considered low risk, potential biases remained. Among all included articles, only two specified the randomization method: one used weight as a baseline characteristic, and one employed a random number table. Twenty-eight studies reported a random housing process. All 29 studies were considered high risk regarding randomization and blinding, with insufficient details provided for assessing baseline characteristics, random outcome assessment, blinding of outcome assessment, and incomplete outcome data. All included studies fully reported expected results, and no other biases were identified.

### 3.3 Therapeutic potential and underlying mechanisms of natural products for CB

**FIGURE 4:**
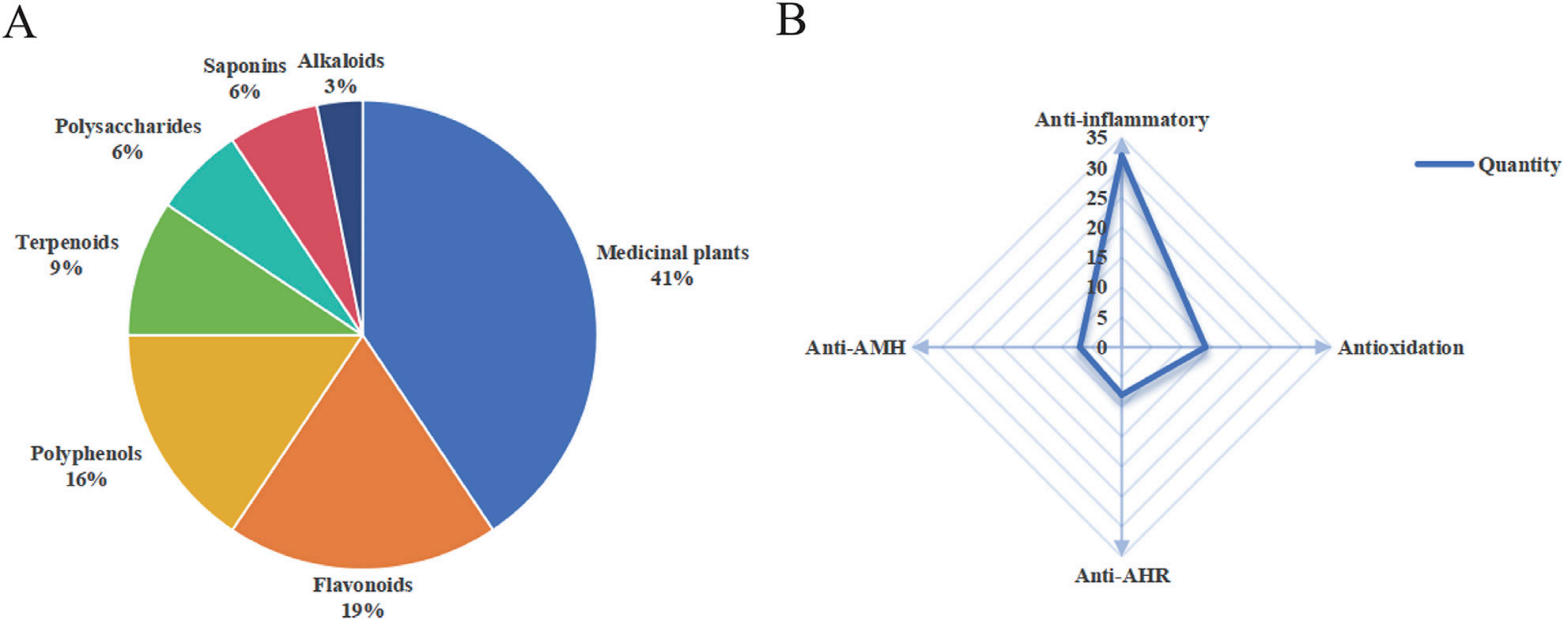
Comprehensive profile of natural products against CB. **(A)** Classification. **(B)** Pharmacological activities. AHR, airway hyperresponsiveness; AMH, airway mucus hypersecretion.

Twenty-nine studies demonstrated that medicinal plants and compounds exhibited anti-inflammatory, antioxidative, antitussive, and expectorant activities against CB. Thirteen medicinal plants and nineteen plant-derived compounds with the aforementioned properties are described alphabetically in the text. Figure 4 presents the classification (A) and pharmacological activities (B) of these natural products, respectively. The scientific names of all medicinal plants were confirmed using the Kew Science database (https://mpns.science.kew.org/). Detailed information is summarized in Tables 1–7.

#### 3.3.1 Medicinal plants against CB

Medicinal plants demonstrating therapeutic potential against CB are presented alphabetically. In numerous studies, their efficacy has been compared with standard clinical medications. These medicinal plants span diverse species, most exerting their effects by regulating immune-inflammatory responses, oxidative stress, bronchospasm, goblet cell metaplasia, and tissue damage repair (Table 1).

##### 3.3.1.1 *Aconitum septentrionale* Koelle

*Aconitum septentrionale* Koelle (*A. septentrionale*; Mongolian name: Gaburidilo) is a mesophytic herb belonging to the Ranunculaceae family. Officially recorded in the third edition of Inner Mongolia Flora, this traditional Mongolian medicine is valued for clearing lung heat and suppressing cough. Ethanol extracts of *A. septentrionale* administered at doses of 0.3, 0.6, and 0.9 mg/kg for 20 consecutive days produced dose-dependent therapeutic effects in a CB model. The high-dose group exhibited efficacy comparable to the positive control (Gejie Dingchuan Capsules; Drug Approval Number: 10940033). The high-dose extract significantly reduced interleukin-1β (IL-1β) levels in lung tissue and inhibited chemokine release (Han et al., 2011).

##### 3.3.1.2 *Ajuga decumbens* Thunb

*Ajuga decumbens* Thunb. (*A. decumbens*; family: Lamiaceae) is a widely distributed herbaceous plant in East Asia, traditionally used in China, Korea, and Japan for its heat-clearing and detoxifying properties (Sun et al., 2012). Due to its anti-inflammatory, antioxidant, antibacterial, and anticancer effects, this plant has a long-standing clinical history in China and was included in the 1977 Chinese Pharmacopoeia (Part I) (Olatunde et al., 2023). Aqueous extracts of *A. decumbens* at doses of 100, 200, and 400 mg/kg exerted dose-dependent therapeutic effects in a CB model. These extracts alleviated bronchial obstruction and lymphocyte infiltration to varying degrees across treatment groups. All dosage groups significantly reduced leukocyte counts in blood and bronchoalveolar lavage fluid (BALF) of the CB model. Medium- and high-dose treatments markedly reduced neutrophil and granulocyte counts in BALF and decreased plasma levels of malondialdehyde (MDA) and myeloperoxidase (MPO). Additionally, these treatments restored activities of antioxidant enzymes (superoxide dismutase [SOD] and glutathione peroxidase [GSH-Px]). The low-dose group only significantly attenuated the reduction in GSH-Px activity, with no statistically significant change in MPO activity (P > 0.05) (P > 0.05) (Xiong et al., 2016).

##### 3.3.1.3 *Atalantia buxifolia* (Poir). Oliv. ex Benth

*Atalantia buxifolia* (Poir.) Oliv. ex Benth (*A. buxifolia*; family: Rutaceae) is a shrub growing in South China, Vietnam, Cambodia, and Laos. This traditional folk medicinal plant is commonly used for treating cough, influenza, and bronchitis (Deng et al., 2008). In a rat model of CB, treatment with ethanol extracts of *A. buxifolia* (2.5 g/kg for 10 days) resulted in significant increases in body weight and alleviation of wheezing and coughing compared to the model control. The extract outperformed the positive control drug (Guilonkechuanning Capsules; 750 mg/kg; Drug Approval Number: 070701) in reducing peribronchial inflammation, interstitial inflammation, and emphysematous changes. It also markedly attenuated epithelial cell proliferation and airway gland thickening. Concurrently, treatment significantly decreased the pro-inflammatory cytokine TNF-α and increased anti-inflammatory cytokines IL-4 and IL-10 levels in lung tissue homogenates (Yin et al., 2014).

##### 3.3.1.4 Bambusae caulis in taeniam

*Bambusae caulis in taeniam* (BC; family: Poaceae), a traditional East Asian medicine with broad therapeutic applications, is derived from the inner green layer of the stems of *Phyllostachys nigra* var. henonis (Mitford) Rendle (Lim et al., 2017). Exhibiting notable anti-inflammatory, antioxidant, anti-allergic, and immunomodulatory properties, BC has been clinically employed in Chinese and Korean traditional medicine to treat various respiratory disorders, including bronchitis and pneumonia (Jiao et al., 2007; Kim et al., 2013; Qi et al., 2009). Compared to the control group, treatment with BC extract (50 and 100 mg/kg) effectively prevented weight loss and significantly reduced total inflammatory cell counts in BALF in a dose-dependent manner. Specifically, BC administration led to reductions in total cells (14.3% and 49.2%), macrophages (19.4% and 59.1%), neutrophils (1.7% and 33.2%), and lymphocytes (28.8% and 55.9%). Furthermore, BC significantly downregulated key pro-inflammatory cytokines, including tumor necrosis factor-alpha (TNF-α) (25.5% and 47.6%), IL-6 (9.1% and 49.0%), and C-C motif chemokine ligand 2 (CCL2) (16.3% and 28.1%). The higher dose (100 mg/kg) markedly suppressed IL-1β expression. BC also alleviated perivascular and peribronchial inflammatory cell infiltration and significantly reduced lung inflammation indexes (Kim et al., 2018).

##### 3.3.1.5 *Citrus grandis* (L.) Osbeck

Exocarpium *Citri grandis* (L.) Osbeck (*C. grandis*; family: Rutaceae), known as “Huajuhong” in Chinese, is the dried epicarp of the fruit of Citrus grandis ‘Tomentosa’. Revered as “Southern Ginseng” for millennia, this traditional Chinese herb exhibits potent antitussive, expectorant, and anti-inflammatory properties (Hu et al., 2025). Compared to the model group, aqueous extract (1,005 mg/kg) and 70% ethanolic extracts (247, 493, and 986 mg/kg) significantly suppressed ammonia-induced cough frequency within 3 min, with inhibition rates of 25.76%, 15.17%, 40.56%, and 43.53%, respectively. Additionally, the 493 mg/kg ethanolic extract markedly prolonged cough latency by 32.57%. All extracts significantly increased tracheal phenol red secretion (by 27.78%, 36.11%, 100%, and 116.67%), indicating enhanced expectorant efficacy, and ameliorated xylene-induced ear edema (with inhibition rates of 23.68%, 42.11%, 59.21%, and 73.68%), demonstrating anti-inflammatory activity (Jiang et al., 2014).

##### 3.3.1.6 *Elaeagnus pungens* Thunb

*Elaeagnus pungens* Thunb. (*E. pungens*; family: Elaeagnaceae) is widespread in subtropical and temperate regions of East and Southeast Asia. The leaves of *E.* pungens were recorded approximately 430 years ago in the seminal Chinese materia medica “Compendium of Materia Medica” (Bencao Gangmu) compiled during the Ming Dynasty (Sun et al., 2021). Traditionally, it has been employed to manage asthma, cough, and bronchitis due to its antitussive, expectorant, anti-asthmatic, and smooth muscle relaxant properties. Administration of *E. pungens* leaf extracts (2.5 and 5 g/kg) inhibited xylene-induced ear edema in mice by 60.2% and 40.7%, respectively. Compared to the model group (incubation period: 39.7 ± 2.5 s; cough frequency: 66.1 ± 1.9 times/3 min), both treatment groups significantly prolonged the incubation period to 52.6 ± 5.5 s and 54.0 ± 5.3 s and reduced cough frequency to 50.8 ± 2.3 and 51.3 ± 1.9 times/3 min, respectively (Zhu et al., 2018).

##### 3.3.1.7 *Eucalyptus globulus* Labill

*Eucalyptus globulus* Labill. (*E. globulus*; family: Myrtaceae) is extensively employed in Brazilian traditional medicine as a secretolytic agent for respiratory infections, including bronchitis, influenza, cough, and sinusitis. *Eucalyptus globulus* oil administered at doses of 30, 100, and 300 mg/kg significantly reduced the total cell count in BALF. Regarding differential cell counts, the low-dose group markedly decreased neutrophil numbers, while the high-dose group significantly reduced macrophages, neutrophils, and lymphocytes. Furthermore, high-dose treatment notably downregulated the expression of Mucin-5AC (MUC5AC) in BALF and decreased both the absorbance and positively stained area of MUC5AC in the tracheobronchial epithelium. Additionally, *E. globulus* oil effectively ameliorated lipopolysaccharide (LPS)-induced bronchiolitis and interstitial pneumonia, as evidenced by histological improvements (Lu et al., 2004).

##### 3.3.1.8 *Gentiana veitchiorum* Hemsl

*Gentiana veitchiorum* Hemsl. (family: Gentianaceae; Tibetan name: Bangjian) is a perennial herb indigenous to forest edges and alpine grasslands of central and eastern Tibet (Wang et al., 2024). Historically, this traditional Tibetan medicine has been used for respiratory ailments owing to its anti-inflammatory, antimicrobial, and detoxification properties. The alcohol extract of *G. veitchiorum* Hemsl. (4 g/kg) significantly downregulated serum levels of the pro-inflammatory cytokine TNF-α and upregulated the anti-inflammatory cytokine IL-10 in an ammonia-induced CB model. Histopathological examination showed that improvements induced by the extract were comparable to the positive control drug, GuiLong KeChuanNing Capsules (1 g/kg). Furthermore, the treatment markedly enhanced antioxidant capacity, evidenced by increased levels of SOD and total antioxidant capacity (T-AOC) (Geng et al., 2010).

##### 3.3.1.9 *Glycyrrhiza uralensis* Fisch. ex DC

*Glycyrrhiza uralensis* Fisch. ex DC. (*G. uralensis*; family: Fabaceae), commonly known as licorice, is an effective herbal medicine widely employed for respiratory diseases in China due to its lung-moistening and cough-relieving effects (Yang et al., 2025). *Glycyrrhiza uralensis* extracts and their secondary metabolites possess diverse biological activities, including anti-inflammatory, antioxidant, and antiviral properties. Administration of an aqueous extract of *G. uralensis* (10 mg/kg, b.i.d.) significantly attenuated inflammatory and oxidative responses in a mouse model of cigarette smoke-induced CB. Compared to the model group, treated animals exhibited marked alleviation of pathological injury in tracheal and pulmonary tissues, along with restoration of oxidative-antioxidative balance. This improvement was evidenced by decreased MDA levels and increased SOD activity in lung tissue homogenates (Chen et al., 2007).

##### 3.3.1.10 *Inula japonica* Thunb

*Inula japonica* Thunb. (*Inula japonica*; family: Asteraceae), known as “Xuanfuhua” in Chinese, is a widely utilized ethnomedicine in Asia, Europe, and North America for bronchitis, inflammation, and digestive disorders. Modern pharmacological research confirms its anti-inflammatory, antioxidant, antiallergic, and anti-pulmonary fibrosis properties (Yang et al., 2016). Ma et al. reported that alcoholic extracts of *I. japonica* (0.25, 0.5, and 1 g/kg) ameliorated pulmonary inflammation and oxidative stress in a rat model of CB induced by LPS and cigarette smoke exposure. The extract significantly attenuated inflammatory cell infiltration and pathological alterations in airways and pulmonary architecture, with the high-dose group (1 g/kg) exhibiting the strongest effects. Additionally, high-dose treatment markedly reduced inflammatory cell counts in BALF and significantly decreased serum levels of pro-inflammatory mediators (IL-6, IL-17, PGE2, COX-2, AP-1), while increasing anti-inflammatory cytokine IL-10. The extract concurrently reduced oxidative stress markers (NO and iNOS levels). All treated groups also significantly improved key pulmonary function parameters (peak inspiratory flow [PIF], peak expiratory flow [PEF], and forced expiratory flow at 50% of FVC [FEF_50_]) (Ma, 2023).

##### 3.3.1.11 *Lysionotus pauciflorus* Maxim

*Lysionotus pauciflorus* Maxim. (*Lysionotus pauciflorus*; family: Gesneriaceae), known as “Shidiaolan” in Chinese medicine, is an important ethnopharmacological herb among the Miao people for managing CB (Takizawa et al., 2025). This medicinal plant exhibits multi-target pharmacological activities, including antitussive, anti-inflammatory, and antimycobacterial effects (Liang et al., 2018). Zhao et al. reported that administration of an aqueous extract of *L. pauciflorus* (2 and 8 g/kg) for 28 days significantly attenuated pulmonary inflammation in a rat model of CB induced by LPS and cigarette smoke, with efficacy comparable to the positive control drug (GuiLong KeChuanNing, 1 g/kg). Treatment improved pathological alterations such as alveolar wall thickening, inflammatory cell proliferation, and luminal secretion, and significantly reduced lung tissue TLR4 mRNA expression (Zhao et al., 2018).

##### 3.3.1.12 *Phyllanthus emblica* L

*Phyllanthus emblica* L. (*P. emblica* L., family: Phyllanthaceae), indigenous to tropical Southeast Asia, is valued for the pharmacological properties of its fruits, leaves, and flowers (Qu et al., 2025). The Chinese Pharmacopoeia (2020) officially documents its functions in clearing lung heat and resolving phlegm, with clinical indications including bronchitis, asthma, and cough. Zhong et al. demonstrated that administration of the ethyl acetate extract from *P. emblica* L. leaves (5, 10, and 15 mg/kg) significantly improved arterial blood gas parameters in a rat model of CB induced by LPS and cigarette smoke. Specifically, the extract increased arterial partial pressure of oxygen (PaO_2_) from 58.6 ± 8.9 mmHg (model group) to 91 ± 5.0 mmHg, and decreased arterial partial pressure of carbon dioxide (PaCO_2_) from 45.1 ± 5.7 mmHg to 40.8 ± 3.6 mmHg. Furthermore, the extract exhibited notable anti-inflammatory effects, demonstrated by reduced pathological scores for bronchiolar inflammation and alveolar damage, decreased total cell counts, and lower numbers of lymphocytes, macrophages, and neutrophils in BALF (Zhong and Zeng, 2008).

##### 3.3.1.13 *Rohdea fargesii* var. *fargesii*

*Rohdea fargesii* var. *Fargesii* (*R. fargesii* var. *fargesii,* family: Asparagaceae), known as “Kai-Kou-Jian” in Chinese, is recognized in southern China for its anti-inflammatory properties (Xu et al., 2007). Compared to the model group, extracts of *R. fargesii* var. *fargesii* (100 and 500 mg/kg) effectively ameliorated pathological features in a cigarette smoke-induced CB model. The treatment significantly reduced cough frequency induced by ammonia within 3 min and prolonged the cough latency period. The high-dose group notably enhanced sputum expectoration, indicated by increased phenol red secretion in the trachea. Both dosage groups significantly suppressed auricular swelling (inhibition rates: 46.04% and 60.99%). Additionally, the extracts substantially decreased serum levels of inflammatory cytokines (TNF-α and IL-6) and reduced inflammatory cell infiltration in lung tissue and luminal exudate in the airways (Li, 2008).

#### 3.3.2 Plant-derived compounds against CB

Researchers have sought to isolate bioactive compounds from medicinal plants demonstrating efficacy in CB management. These phytoconstituents are classified into six major categories: flavonoids, polyphenols, terpenoids, glycosides, saponins, and alkaloids. For each category, we provide a systematic overview of their therapeutic properties and discuss their associated mechanisms of action based on CB animal studies.

##### 3.3.2.1 Flavonoids

Flavonoids, a major class of plant secondary metabolites, possess a characteristic C6–C3–C6 skeletal structure consisting of two aromatic rings connected by a three-carbon chain. These compounds are ubiquitously present in diverse plant species and exhibit a broad spectrum of pharmacological activities, including anti-inflammatory, antibacterial, antiviral, antioxidant, and analgesic effects. Accumulating evidence indicates that flavonoids hold therapeutic potential against CB, primarily through modulation of key inflammatory signaling pathways and interaction with multiple molecular targets implicated in CB pathogenesis (Table 2).

Flos Inulae flavonoid, a natural compound derived from *I*. *japonica*, exhibits diverse pharmacological properties, including anti-inflammatory, antioxidant, and anti-AHR effects. Ma (2023) demonstrated that treatment with Flos Inulae flavonoid (0.005 g/kg) significantly alleviated airway inflammation in a rat model of CB induced by cigarette smoke exposure and LPS challenge. After 56 days of continuous administration, the compound effectively reduced inflammatory cell infiltration, downregulated pro-inflammatory mediators (IL-6, COX-2, AP-1, and iNOS), and upregulated the anti-inflammatory cytokine IL-10. Additionally, it improved AHR parameters compared to the untreated model control group. Although a decreasing trend in the inflammatory cell count in BALF was observed, this change was not statistically significant (Ma, 2023).

Kaempferol is a natural flavonol found in various plants and plant-derived foods, including kale, beans, tea, spinach, and broccoli (Rajizadeh et al., 2024). Zhu et al. reported that administration of kaempferol from *E. pungens* (0.05 or 0.1 g/kg for 5 days) significantly attenuated the progression of CB compared to the model group. Specifically, kaempferol demonstrated inhibitory effects on inflammatory edema (65.5% and 44.2%), exhibited analgesic effects (59.9% and 63.1%), and reduced cough frequency from 66.1 ± 1.9 to 49.7 ± 2.1 and 35.2 ± 4.0 times/3 min, respectively (Zhu et al., 2018).

Naringin, a colorless and flavorless flavanone glycoside, is naturally found in grapes, citrus fruits, and the traditional Chinese herbal medicine Huajuhong, with demonstrated efficacy in improving respiratory disorders (Zhang et al., 2025). In a Hartley guinea pig model of cigarette smoke-induced CB, naringin was administered orally (9.2, 18.4, and 36.8 mg/kg) once daily for 56 days, administered 1 h prior to cigarette smoke exposure. The study demonstrated significant reductions in total inflammatory cell counts and numbers of leukocytes, neutrophils, lymphocytes, monocytes, eosinophils, and basophils, as well as decreased production of IL-8, TNF-α, and Leukotriene B4 (LTB4), and elevated levels of Lipoxin A4 (LXA4) in BALF. Furthermore, naringin significantly suppressed MPO activity in both BALF and lung tissues while enhancing SOD activity in lung tissue. Additionally, the intervention demonstrated dose-dependent therapeutic effects, evidenced by reduced airway resistance and decreased cough frequency within a 10-min observation period (Luo et al., 2012).

Quercetin (3,3′,4′,5,7-pentahydroxyflavone), a bioactive flavonoid abundant in various plants such as *E. pungens*, berries, and tomatoes, has demonstrated significant antitussive, anti-inflammatory, and antioxidant properties across experimental models (Dong et al., 2025). A pharmacological study revealed that quercetin administered at doses of 0.05 and 0.1 g/kg for 5 days exhibited antitussive efficacy comparable to pentoxyverine citrate (0.01 g/kg), markedly reducing cough frequency from 66.1 ± 1.9 to 43.0 ± 3.2 and 38.2 ± 4.2 times per 3 min, and inhibiting xylene-induced ear swelling by 38.1% and 46.7%, respectively (Zhu et al., 2018). Additionally, Yang et al. confirmed that quercetin at doses of 25 and 50 mg/kg significantly alleviated pathological lung injury, reduced total and differential cell counts in BALF, and decreased levels of IL-8 and TNF-α. These effects were mediated by inhibiting activation of the NF-κB signaling pathway, demonstrating its anti-inflammatory activity. Furthermore, quercetin significantly increased lung levels of GSH and T-AOC, indicating enhanced antioxidant activity. Regarding anti-AMH effects, quercetin reduced intracellular mucous glycoconjugate production and decreased both mRNA and protein expression of MUC5AC. Additionally, it significantly suppressed phosphorylated EGFR (p-EGFR) protein expression (Yang et al., 2012).

Scutellarin (4′,5,6-trihydroxyflavone-7-glucuronide), a primary active constituent of medicinal plants such as *Scutellaria altissima* L., *Scutellaria baicalensis* Georgi and *Scutellaria barbata* D. Don, has exhibited notable anti-inflammatory, antioxidant, and neuroprotective activities in various experimental studies (Tang et al., 2025). Wang et al. reported that scutellarin (40 mg/kg) significantly reduced lung pathological scores and the wet/dry (W/D) weight ratio of the right lung in a rat model of CB induced by LPS and cigarette smoke. Concurrently, it markedly decreased protein content, leukocyte counts, and neutrophils in BALF. Treatment also significantly downregulated the expression of IL-1β and IL-6 in BALF, reduced IL-17A and TNF-α in serum, and upregulated IL-10 levels. Furthermore, scutellarin exhibited potent antioxidant activity, significantly reducing MDA and increasing SOD levels in lung tissue homogenates. Additionally, it significantly suppressed both mRNA and phosphorylated protein expression of PI3K, AKT, and mTOR (Wang et al., 2023a).

Seabuckthorn flavone, a principal bioactive component of *Hippophae rhamnoides* L., has demonstrated significant anti-inflammatory properties (Mishra et al., 2008). According to Ren et al., seabuckthorn flavone at doses of 100, 200, and 500 mg/kg effectively suppressed chronic airway inflammation induced by cigarette smoke combined with LPS. These effects were achieved by reducing leukocyte, neutrophil, and macrophage counts in BALF, and by downregulating pro-inflammatory cytokines (IL-1β, IL-6, COX-2, CXCL1) and the mucus marker protein MUC5AC. Additional *in vitro* experiments within the same study suggested the anti-CB effects of seabuckthorn flavone may be mediated through modulation of key signaling pathways, including Extracellular Signal-Regulated Kinase (ERK), Akt, and Protein Kinase C (PKC) (Ren, 2019).

##### 3.3.2.2 Polyphenols

Polyphenols represent a diverse class of phytochemicals widely distributed in plant-based foods and medicinal botanicals, particularly concentrated in fruits, bark, roots, and leaves (Zamani-Garmsiri et al., 2022). Owing to their bioactive properties, these compounds have emerged as promising therapeutic agents because of their dual capacity to mitigate oxidative stress and inflammatory responses. Current pharmacological research has identified four specific polyphenols demonstrating significant efficacy in ameliorating CB in animal models through these complementary mechanisms (Table 3).

Bergenin, a bioactive polyphenol isolated primarily from *Bergenia purpurascens* (Hook.f. and Thomson) Engl., has been traditionally employed in Chinese Miao medicine for respiratory disorders. This compound exhibits prominent anti-inflammatory, antioxidant, antitussive, and expectorant properties (Yang et al., 2017). Compared to the cigarette smoke-induced CB model group, administration of bergenin (87.5 mg/kg) significantly reduced leukocyte counts in BALF from (2.51 ± 0.14) × 10^5^/mL to (1.55 ± 0.13) × 10^5^/mL, macrophage counts from (0.99 ± 0.13) × 10^5^/mL to (0.62 ± 0.07) × 10^5^/mL, neutrophil counts from (0.91 ± 0.08) × 10^5^/mL to (0.35 ± 0.02) × 10^5^/mL, and lymphocyte counts from (0.20 ± 0.02) × 10^5^/mL to (0.08 ± 0.01) × 10^5^/mL. Furthermore, bergenin treatment markedly attenuated pathological alterations in lung tissues, including inflammatory cell infiltration and mucus hypersecretion (Zhang et al., 2019).

*p*-Coumaric acid (4-hydroxycinnamic acid, *p*-CA), a bioactive phenolic compound derived from *P. nigra* var. henonis and widely distributed in fruits, vegetables, and plant-derived products, has attracted significant research interest due to its diverse pharmacological properties. These include potent anti-inflammatory, antioxidant, antibacterial, and antiglycation activities (Elrherabi et al., 2024). In a cigarette smoke-induced CB model, Kim et al. reported that treatment with *p*-CA (5 and 10 mg/kg) significantly reduced inflammatory cell infiltration around bronchial and vascular regions. Additionally, *p*-CA administration markedly suppressed the production of several key inflammatory mediators, reducing IL-6 by 10.2% and 49.0%, TNF-α by 51.6% and 37.1%, and CCL2 by 70.3% and 67.1%, respectively. Notably, the reduction in CCL2 levels achieved with *p*-CA was comparable to the roflumilast-treated positive control group. Furthermore, *p*-CA treatment significantly reduced total and differential inflammatory cell counts in BALF. Total cell numbers declined by 28.6% and 22.2%, neutrophils by 73.6% and 64.3%, macrophages by 40.9% and 19.4%, and lymphocytes by 54.6% and 23.1%. Importantly, pretreatment with p-CA at 5 mg/kg also inhibited nuclear translocation of the NF-κB p65 subunit to an extent similar to roflumilast (Kim et al., 2018).

Polydatin (3,4′,5-trihydroxystilbene-3-β-D-glucoside), a bioactive stilbenoid isolated from *Reynoutria japonica* Houtt. and present in grapes and peanuts, has attracted considerable scientific attention due to its broad pharmacological properties, including antitussive, expectorant, anti-asthmatic, antibacterial, and antioxidant effects (Zhao and Wang, 2025). In a rat model of LPS/cigarette smoke-induced CB, Polydatin (60 mg/kg) ameliorated lung injury by reducing pathological scores, pulmonary edema, and inflammatory cell infiltration in BALF. It strongly suppressed pro-inflammatory cytokines (IL-1β, IL-6, IL-17A, and TNF-α) and upregulated the anti-inflammatory mediator IL-10. Polydatin also exerted antioxidant effects by modulating MDA and SOD levels. The suppression of PI3K/AKT/mTOR pathway activation underlies Polydatin’s efficacy in alleviating CB (Wang et al., 2023a).

Punicalagin, a polyphenol extracted from the dried peel of *Punica granatum* L., is a rich source of antioxidants. It exhibits various pharmacological effects, including anti-inflammatory, antioxidant, antibacterial, and antitumor properties (Varmaghani et al., 2021). A study using a cigarette smoke-induced CB rat model reported that punicalagin (20 and 40 mg/kg) suppressed serum levels of TNF-α, IL-8, IL-1β, and IL-6. It also significantly enhanced the activity of antioxidant enzymes (SOD, GSH-Px, and CAT) and reduced MDA levels in serum and lung tissues. Moreover, punicalagin activated the peroxisome proliferator-activated receptor γ (PPARγ)/nuclear factor-erythroid 2 related factor 2 (Nrf2) antioxidant pathway and increased protein expression of PPARγ, peroxisome proliferator-activated receptor γ coactivator 1α (PGC-1α), Nrf2, and γ-glutamylcysteine synthetase (γ-GCS) (Shang et al., 2017).

Mangiferin, a natural polyphenolic compound and the primary bioactive constituent of *Mangifera indica* L. leaves, demonstrates significant therapeutic efficacy and favorable safety in alleviating CB symptoms, including cough, sputum production, wheezing, and fever (Lawrence et al., 2025). In a cigarette smoke-induced CB rat model, mangiferin administered at 200 mg/kg for 4 weeks showed efficacy comparable to prednisone (5 mg/kg), significantly reducing lung histopathological scores and serum inflammatory markers (C-reactive protein and TNF-α). Mangiferin also markedly downregulated NF-κB p65 mRNA and protein expression while upregulating IκBα. However, at a dose of 100 mg/kg, no significant differences occurred compared to the model group, indicating the anti-inflammatory effects of mangiferin in CB are dose-dependent (Wei et al., 2014).

##### 3.3.2.3 Terpenoids

Terpenoids consist of five-carbon isoprene units (C5H8) and constitute the largest and most diverse group of natural plant compounds. They possess various medicinal functions, including antioxidant and anti-inflammatory effects (Wang et al., 2023b). Several terpenoid-rich plants such as *Cannabis sativa*, *Artemisia annua*, *Salvia miltiorrhiza*, *Ginkgo biloba*, and *Taxus media* exhibit notable therapeutic potential. This section systematically reviews the mechanisms by which terpenoids modulate key inflammatory mediators and signaling pathways related to CB pathogenesis (Table 4).

Sesquiterpenes, bioactive constituents of Flos Inulae (Asteraceae family), demonstrate diverse pharmacological activities, including anti-inflammatory, antioxidant, anticancer, and antibacterial effects (Chen et al., 2024; Niu et al., 2024). Ma et al. reported that administration of Flos Inulae sesquiterpene (0.03 g/kg) improved lung function and reduced oxidative marker iNOS in a rat model of LPS and cigarette smoke-induced CB. Treatment alleviated inflammation by decreasing inflammatory cell counts in BALF, lowering serum levels of pro-inflammatory cytokines (IL-6, IL-17), and suppressing COX-2 expression. It also downregulated the protein levels of NF-κB p65 and IκBα in lung tissues and concurrently increased anti-inflammatory cytokineIL-10 (Ma, 2023).

Glycyrrhizin, a bioactive triterpenoid saponin from *G*. *uralensis*, has potent anti-inflammatory properties. Wang et al. demonstrated that glycyrrhizin (2.5, 5.0, and 10.0 mg/kg) administered over 30 days significantly reduced both protein and mRNA expression of TNF-α and IL-1β in a dose-dependent manner. The greatest inhibitory effect occurred in the high-dose group, where cytokine levels approached normal values (Wang and Chen, 2016).

Ursolic acid (3beta-Hydroxyurs-12-en-28-oic acid), a pentacyclic triterpenoid isolated from *Eriobotrya japonica* (Thunb.) Lindl. (TAL), exhibits therapeutic potential for chronic airway diseases through its anti-inflammatory and antioxidant properties (Luan et al., 2022). Huang et al. reported that TAL (50, 150, and 450 mg/kg) administered for 2 weeks after exposure to LPS and *bacillus* Calmette-Guérin significantly reduced several oxidative mediators, including HO-1 expression, corresponding mRNA, and MDA production, while increasing SOD expression (Huang et al., 2006). In the same model, TAL also significantly inhibited elevations in NO, iNOS concentration, iNOS activity, and associated mRNA and protein expression. Furthermore, it reduced p38 MAPK phosphorylation in CB rats (Huang et al., 2009). Additional studies confirmed TAL’s dose-dependent attenuation of inflammation through suppression of TNF-α, IL-1, PGE2, and LTB4, and inhibition of NF-κB p65 activation (Huang et al., 2007). Ge et al. demonstrated that TAL significantly decreased counts of leukocytes, neutrophils, and alveolar macrophages in BALF, lowered IL-8 and TNF-α levels in lung homogenates, reduced NF-κB and intercellular adhesion molecule-1 (ICAM-1) expression on bronchial epithelium, and simultaneously increased IL-10 levels (Ge et al., 2009). Further evidence suggests TAL’s anti-inflammatory effects involve the ERK and p38 pathways, as inhibitors of these kinases significantly suppressed TNF-α release (Jiang et al., 2009).

##### 3.3.2.4 Polysaccharides

Polysaccharides are carbohydrate polymers composed of ten or more monosaccharide units linked by glycosidic bonds. As bioactive components of medicinal plants, they possess multiple pharmacological properties, including anti-inflammatory, antioxidant, antibacterial, and antiviral activities. Polysaccharides act as non-specific immunomodulators and serve as potential sources for new drug development. Two specific polysaccharides effectively inhibit inflammatory, oxidative, and immune reactions in CB (Table 5).

Polysaccharides from *I.* japonica exhibit significant therapeutic effects, such as relieving cough, resolving phlegm, anti-inflammatory, antioxidant, and antibacterial properties. Ma et al. (2023) reported that administration of Flos Inulae polysaccharides (0.17 g/kg) partially improved lung function and attenuated histopathological changes in lung tissue. Additionally, it significantly reduced serum pro-inflammatory cytokine IL-6 levels and elevated anti-inflammatory factors in rats with cigarette smoke and LPS-induced CB (Ma, 2023).

Polysaccharides also play a crucial role in the clinical application of *Platycodon grandiflorus* (Jacq.) A. DC. (Campanulaceae) for CB treatment by modulating pulmonary immune responses (Sun et al., 2025). Liu et al. demonstrated that polysaccharides extracted from *P. grandiflorus* (75, 150, and 300 mg/kg daily) mitigated inflammation in a sulfur dioxide-induced CB rat model after 15 days of treatment. The anti-inflammatory mechanism involved reduced Th1-type immune responses, evidenced by decreased CD4^+^IFN-γ^+^ T cell counts, lower IFN-γ levels, and suppressed TNF-α protein expression. Simultaneously, enhanced Th2-type responses occurred, characterized by increased CD4^+^IL-4^+^ T cell populations and elevated IL-4 expression. Furthermore, polysaccharides demonstrated antioxidant effects by inhibiting ET-1, NO, and iNOS production, and anti-mucus effects by downregulating MUC2 protein expression (Liu et al., 2022).

##### 3.3.2.5 Saponins

Saponins are naturally occurring plant compounds classified as either steroid or triterpenoid glycosides. Widely distributed in plants, they exhibit diverse biological functions, particularly anti-inflammatory and antioxidant activities. In CB, saponins have shown significant anti-inflammatory potential (Table 6).

Kikyosaponin, a bioactive triterpenoid saponin derived from *P. grandiflorus*, exhibits therapeutic potential in alleviating airway inflammation in CB (Yim et al., 2016). He et al. reported that daily administration of kikyosaponin (12.5, 25.0, and 50.0 mg/kg) for 30 days markedly improved lung pathology in a cigarette smoke- and ammonia liquor-induced CB mouse model. Treatment significantly thinned the bronchial wall, enlarged the airway lumen, and markedly reduced inflammatory cell infiltration. Additionally, kikyosaponin dose-dependently reduced TNF-α and IL-1β protein expression levels in lung tissues, thus preventing airway inflammation (He et al., 2013).

Platycodin D, a saponin isolated from the root of *P. grandiflorus*, exhibits potent activity against CB (Song et al., 2025). Liu et al. reported that administration of Platycodin D (2 mg/kg) for 15 days alleviated airway inflammation in a sulfur dioxide-induced CB rat model. This effect was mediated by reduced Th1 immune responses, evidenced by decreased CD4^+^IFN-γ^+^ cell counts and lower IFN-γ and TNF-α levels, alongside enhanced antioxidant capacity. Concurrently, Th2 responses increased, indicated by elevated CD4^+^IL-4^+^ cell proportions and higher IL-4 expression. Moreover, Platycodin D effectively suppressed mucus hypersecretion by downregulating MUC2 protein expression (Liu et al., 2022).

##### 3.3.2.6 Alkaloids

Alkaloids, nitrogen-containing basic organic compounds primarily derived from plants, have high biological activity and represent key active ingredients in traditional Chinese medicine. This review highlights one alkaloid that modulates inflammatory responses in CB (Table 7).

Aconitum total alkaloids possess potent analgesic, anti-inflammatory, antitussive, and immunomodulatory properties (Şuţan et al., 2024). Hong et al. demonstrated that aconitum total alkaloids (50 and 100 mg/kg) markedly reduced bronchial inflammatory cell infiltration and decreased pro-inflammatory cytokines (TNF-α, IL-1, and IL-8) in a LPS and BCG-induced CB rat model. The anti-inflammatory efficacy of aconitum total alkaloids was comparable to that of dexamethasone (1.2 mg/kg) (Hong et al., 2019).

## 4 Discussion

**FIGURE 5:**
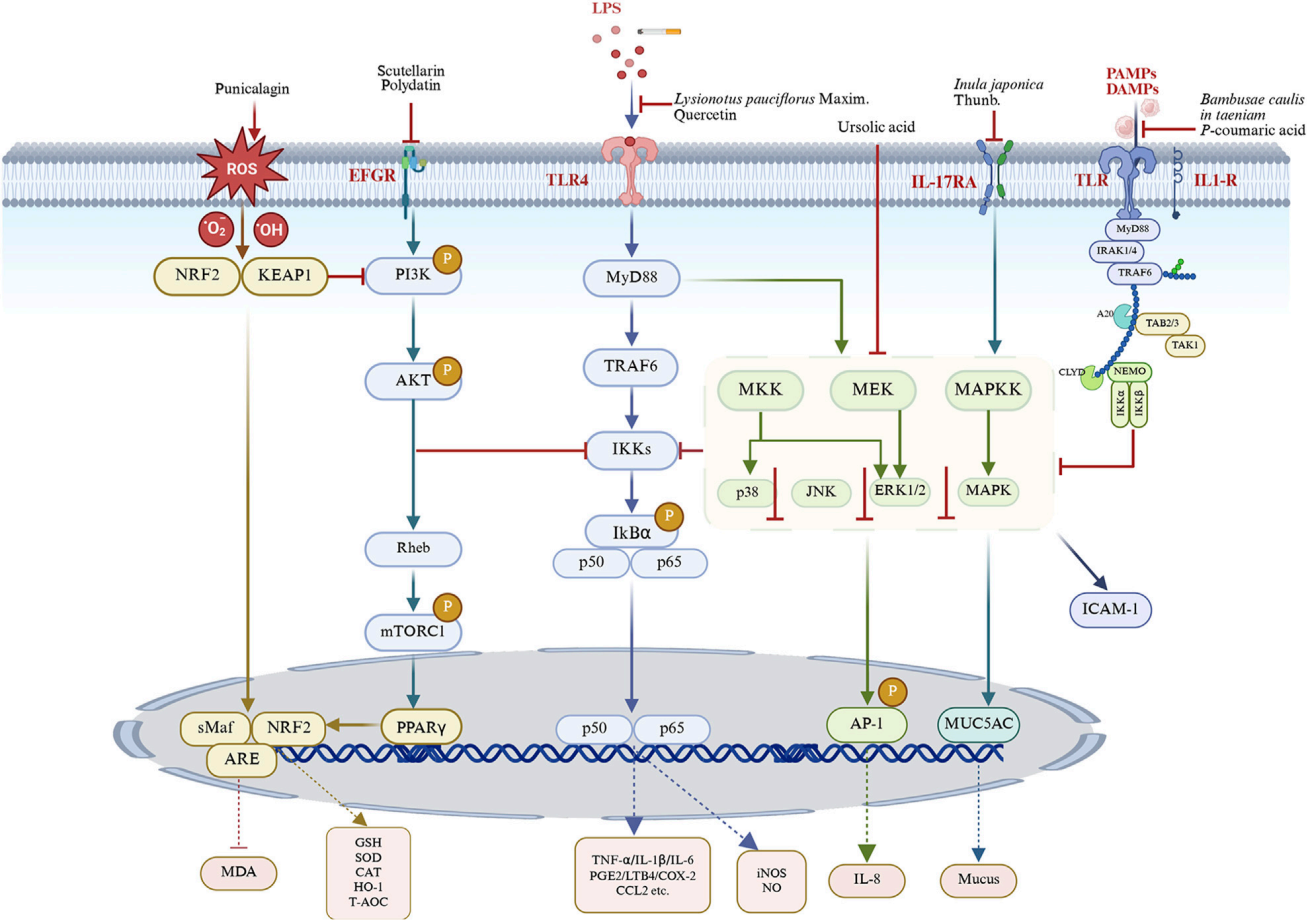
Unraveling the signaling mechanisms of natural products against CB. (BioRender number: DP28SPPAEG). By modulating key inflammatory pathways such as NF-κB, PI3K/AKT/mTOR, TLR4, MAPK, and Nrf2 pathways, medicinal plants and their constituents can effectively mitigate inflammatory responses, oxidative damage, mucus hypersecretion and airway hyperreactivity. LPS, lipopolysaccharides; TLR4, toll-like receptor 4; MyD88, myeloid differentiation primary response gene 88; TRAF6, tumor necrosis factor receptor-associated factor 6; IKK, IκB kinase; PAMPs, pathogen-associated molecular patterns; DAMPs, damage-associated molecular patterns; PI3K, phosphatidylinositol 3-kinase; AKT, protein kinase B; mTOR, mechanistic target of rapamycin; Keap1, kelch-like ECH-associated protein 1; NRF2, nuclear factor erythroid-2-related factor 2; sMaf, small Musculoaponeurotic Fibrosarcoma; ARE, antioxidant Response Element; MDA, malonaldehyde; GSH, glutathione; SOD, superoxide Dismutase; CAT, catalase; HO-1, heme oxygenase-1; T-AOC, total -antioxidant capacity; ERK, extracellular signal-related kinase; MKK, MAP kinase kinases; MAPKK, MAP kinase; MEK, mitogen-activated extracellular signal-regulated kinase; JNK, jun n-terminal kinase; AP-1, activator protein 1; PPARγ, peroxisome proliferator-activated receptor γ; IκBα, inhibitor of kappa B; IKK, IκB kinase; TNF-α, tumor necrosis factor alpha; IL-X, interleukin X; NO, nitric oxide; iNOS, inducible nitric oxide synthase; PGE2, prostaglandin E2; COX-2, cyclooxygenase-2; LTB4, leukotriene B4; CCL2, C-C motif chemokine ligand 2; ICAM-1, intercellular adhesion molecule-1.

CB is a common and heterogeneous disease leading to accelerated lung function decline, worsening airflow obstruction, and increased mortality (Kim and Criner, 2013). Its pathology involves persistent airway inflammation, oxidative stress, hyperresponsiveness, excessive mucus secretion, and bronchial smooth muscle hypertrophy and hyperplasia. Recent studies indicate that various medicinal plants and their compounds significantly alleviate these pathological processes. This review systematically summarizes relevant animal studies, highlighting natural agents as alternative therapies and their associated mechanisms (Figure 5).

Chronic inflammation, the core pathophysiology of CB, involves immune cell activation and inflammatory mediator release through multiple pathways. NF-κB is a critical transcriptional regulator modulating inflammatory responses and innate immunity (Yang et al., 2022). Under basal conditions, NF-κB is inactive in the cytoplasm due to binding with its inhibitory protein, IκBα. Upon cellular stimulation, IκBα undergoes degradation, leading to the release and nuclear translocation of the p50-p65 heterodimer. Within the nucleus, this complex binds specific promoter sequences, activating transcription of pro-inflammatory and inflammation-related genes (Jin et al., 2012). The subsequent production of numerous inflammatory mediators forms a critical link between NF-κB activation and tissue damage. Although essential for immunity, prolonged activation during systemic inflammation can cause lung injury (Jasemi et al., 2022). The resulting inflammatory mediators activate innate and adaptive immunity, recruiting and dysregulating immune cells (macrophages, neutrophils, and lymphocytes), thus promoting chronic airway inflammation (Barnes, 2017). Targeting this pathway, our study demonstrated that quercetin, mangiferin, Flos Inulae sesquiterpene, and ursolic acid exert significant anti-inflammatory effects in rodent models. These compounds inhibited NF-κB signaling, decreased inflammatory cell counts in BALF, and reduced pro-inflammatory cytokines (TNF-α, IL-1β, IL-8, LTB4, and PGE2), while significantly elevating anti-inflammatory mediator IL-10 (Figure 5; Tables 1–4).

The overactivation of the PI3K/AKT/mTOR pathway promotes sustained inflammation and disease progression by regulating inflammatory cell activation, mediator release, and airway remodeling (Bozinovski et al., 2006). Activated PI3K initiates signaling by phosphorylating phosphatidylinositol diphosphate to generate phosphatidylinositol 3,4,5-triphosphate, which recruits Akt and phosphoinositide-dependent kinase-1 to the plasma membrane, leading to Akt activation (Manning and Toker, 2017; Manning and Cantley, 2007). Akt then activates mTOR, a key effector promoting protein synthesis and cellular growth (He et al., 2020). Consistent with PI3K/AKT’s documented role in airway inflammation, treatment with Scutellarin and Polydatin significantly reduced inflammation, as evidenced by decreased serum levels of pro-inflammatory cytokines (TNF-α and IL-17A) and lower levels of IL-6 and IL-1β in BALF (Figure 5; Tables 2 and 3).

Toll-like receptors (TLRs), pattern recognition receptors (PRRs) of the innate immune system, form a first line of defense by recognizing pathogen-associated molecular patterns (PAMPs) and damage-associated molecular patterns (DAMPs), initiating immune responses (Richards et al., 2002). However, under pathological conditions, hyperactivated TLRs exacerbate inflammation (Wu et al., 2018). Except for TLR3, most TLRs depend on the adaptor protein MyD88 to activate critical inflammatory pathways, including NF-κB, MAPK, and type I interferon, ultimately triggering cytokine, chemokine, and protease release (Miller et al., 2019). We found that *L. pauciflorus* Maxim exerted protective effects against lung tissue injury by inhibiting the TLR4 signaling pathway (Figure 5; Table 1).

The MAPK pathway is critical in CB pathogenesis, regulating inflammation, cell growth, and apoptosis (Jiang et al., 2022). It comprises three major serine/threonine kinase subfamilies: MAPK14 (p38), c-jun amino-terminal kinase (JNK), and extracellular signal-regulated kinase (ERK) (Johnson and Lapadat, 2022). Simultaneous activation of multiple kinases may initiate the MAPK cascade. Accumulating evidence confirms MAPK’s role in Th2 inflammation and suggests involvement in COPD exacerbations (Lau et al., 2012). Our research demonstrated that ursolic acid ameliorates lung injury and inflammation in CB by inhibiting the MAPK pathway (Figure 5; Table 4).

Oxidative stress, a primary driver of CB progression and exacerbation, amplifies inflammation. It arises from increased inhaled oxidants and elevated reactive oxygen species (ROS) production by inflammatory cells (Son et al., 2020; Kirkham and Rahman, 2006). Excess ROS activates signaling pathways involving transcription factors like NF-κB, AP-1, and Nrf2, which regulate antioxidant responses (Ro et al., 2020). Nrf2 activation protects against inflammation and oxidative damage by upregulating antioxidant factors (HO-1, SOD, GSH) and inhibiting the NLRP3 inflammasome, NF-κB, and MAPK pathways (Ng et al., 2014). We found that punicalagin enhances antioxidant capacity and reduces inflammation by regulating the Nrf2 pathway in CB rats. Additionally, natural products such as *A. decumbens*, *G*. *veitchiorum* Hemsl., *G*. *uralensis* Fisch. ex DC., *I*. *japonica*, Flos Inulae flavonoid, naringin, quercetin, scutellarin, polydatin, punicalagin, Flos Inulae sesquiterpene, ursolic acid, platycodon grandiflorus polysaccharide, and platycodin D collectively demonstrated antioxidant activity, synergistically enhancing their anti-inflammatory efficacy in CB (Figure 5; Tables 1–6).

Secondary critical mechanisms include alleviating AMH and AHR, direct consequences of chronic airway inflammation and the pathological foundation of CB (Kim and Criner, 2013). Mucus hypersecretion primarily involves goblet cell metaplasia and impaired mucus clearance. Our research identified two effective groups of natural products: *E. globulus* Labill., quercetin, seabuckthorn flavone, Platycodon grandiflorus polysaccharide, and platycodin D significantly suppressed mucin expression, while *C. grandis* (L.) Osbeck and *R. fargesii* var. *fargesii* markedly enhanced expectoration. Powerful antitussive effects were demonstrated by *C. grandis* (L.) Osbeck, E. pungens, *R. fargesii* var. *fargesii*, naringin, quercetin, and kaempferol from *E. pungens*. Furthermore, notable improvements in pulmonary function indices occurred with *I. japonica* and Flos Inulae sesquiterpene (Tables 1, 2, and 4).

Among the investigated natural products, several have undergone extensive research and clinical evaluation, demonstrating significant efficacy in managing various respiratory conditions. For instance, *A. septentrionale* has advanced to clinical trials for CB. Treatment with this agent effectively reduces inflammation, improves respiratory function, alleviates clinical symptoms, and lowers complication risks, with reported cure rates of 96.3% (Gao, 1989). A systematic review of clinical studies on eucalyptus oil indicated its potential benefits in managing respiratory diseases such as asthma, COPD, pneumonia, and interstitial pneumonia (Li et al., 2023). Reported outcomes include reduced inflammation, improved lung function, symptom relief, and fewer complications. Moreover, a randomized, placebo-controlled, blind clinical trial demonstrated that 1,8-cineole capsules (the primary component of eucalyptus oil) reduced acute exacerbations in COPD patients and improved dyspnea, quality of life, airflow limitation, and overall health status (Worth et al., 2009). A subsequent *post hoc* analysis of another randomized, placebo-controlled, double-blind trial revealed that Myrtol^®^ standardized, containing 1,8-cineole, significantly lowered COPD exacerbation rates and alleviated cough and sputum production (Beeh et al., 2016). Additionally, a clinical trial assessing eucalyptus oil as an adjunct therapy for COVID-19 has been completed (NCT05398965, http://clinicaltrials.gov/). These findings collectively support eucalyptus oil as a potent adjuvant without adverse effects. Quercetin has also been included in multiple clinical trials related to COPD and COVID-19 (NCT03989271, NCT06003270, NCT01708278, NCT05601180, NCT04851821, and NCT05037240, http://clinicaltrials.gov/), which will provide additional observational evidence. Furthermore, two clinical trials evaluating Bergenin tablets and Huajuhong (*C*. *grandis* L. Osbeck) preparations for respiratory conditions have been registered with the Chinese Clinical Trial Registry (ChiCTR2000030804 and ChiCTR2200066636, http://www.chictr.org.cn). Thus, existing evidence strongly suggests that these natural products should be considered priority candidates for clinical translation.

Ultimately, ursolic acid emerged as a standout candidate due to its multi-target properties. By simultaneously targeting both NF-κB and MAPK inflammation pathways and enhancing antioxidant defenses, it significantly improved pulmonary function and histopathological damage in diverse CB animal models. These compelling preclinical data emphasize its substantial potential for clinical development. However, this translational path faces considerable challenges. Current limitations include the lack of validation in high-quality randomized controlled trials (RCTs), alongside insufficient data on its pharmacokinetics, optimal dosing, and long-term safety profile in humans. Future research must prioritize well-designed Phase I/II clinical trials to establish safety and preliminary efficacy in CB patients. Further key objectives include enhancing the bioavailability of this poorly soluble compound, standardizing its extraction protocols, and exploring its effects in combination with standard therapies.

## 5 Study advantages and limitations

This review provides a comprehensive and systematic synthesis of experimentally validated medicinal plants and compounds for CB treatment, highlighting the most clinically promising candidates. However, some limitations remain. Many included studies were of medium quality due to incomplete blinding information, potentially affecting the accuracy of bias assessments. Additionally, the safety and toxicity of these natural products require further investigation. Although all studies included here used animal models similar to human physiology, the effectiveness of these natural agents still needs verification through clinical practice.

## 6 Conclusion

In conclusion, medicinal plants and compounds effectively treat CB by demonstrating anti-inflammatory, antioxidant, antitussive, and anti-mucus activities *via* pathways including NF-κB, PI3K/AKT/mTOR, TLR4, MAPK, and Nrf2. Targeting these promising signaling pathways, ursolic acid emerges as a particularly promising candidate drug. These findings provide valuable insights for the drug discovery process involving natural agents.
