# Supplementary material for: Medicinal plants and compounds for chronic bronchitis treatment: efficacy and action mechanisms
Source: Front Pharmacol. 2025 Oct 29;16:1674079. doi: 10.3389/fphar.2025.1674079 (PMC12616378; doi:10.3389/fphar.2025.1674079)
Supplement: Supplementary file 1 [file Supplementaryfile1.docx]

**PudMed-477**

**P：“Bronchitis, Chronic”[Mesh] 1952条**

**“**Chronic Bronchitis**” 17493 条**

TS=**("Bronchitis, Chronic"**Bronchitis，Chronic**[Mesh]) OR (Chronic Bronchitis)**

**I："Plants"[Mesh] OR** “Plants” OR “Plant” OR **"Plants, Medicinal"[Mesh]** OR “Medicinal Plant” OR “Plant, Medicinal” OR “Medicinal Plants” OR “Medicinal Herbs” OR “Herb, Medicinal” OR “Medicinal Herb” OR “Herbs, Medicinal” OR “Pharmaceutical Plants” OR “Pharmaceutical Plant” OR “Plant, Pharmaceutical” OR “Plants, Pharmaceutical” OR “Healing Plants” OR “Healing Plant” OR “Plant, Healing” OR “Plants, Healing” **1,308,984**

## Scopus-602

**P:**TITLE-ABS-KEY ( "Bronchitis, Chronic" OR "Chronic Bronchitis" ) 21245

**I：**TITLE-ABS-KEY(“Plants” OR “Plant” OR "Plants, Medicinal" OR “Medicinal Plant” OR “Plant, Medicinal” OR “Medicinal Plants” OR “Medicinal Herbs” OR “Herb, Medicinal” OR “Medicinal Herb” OR “Herbs, Medicinal” OR “Pharmaceutical Plants” OR “Pharmaceutical Plant” OR “Plant, Pharmaceutical” OR “Plants, Pharmaceutical” OR “Healing Plants” OR “Healing Plant” OR “Plant, Healing” OR “Plants, Healing”)

**TS=**( TITLE-ABS-KEY ( "Bronchitis, Chronic" OR "Chronic Bronchitis" ) ) AND ( TITLE-ABS-KEY ( "Plants" OR "Plant" OR "Plants, Medicinal" OR "Medicinal Plant" OR "Plant, Medicinal" OR "Medicinal Plants" OR "Medicinal Herbs" OR "Herb, Medicinal" OR "Medicinal Herb" OR "Herbs, Medicinal" OR "Pharmaceutical Plants" OR "Pharmaceutical Plant" OR "Plant, Pharmaceutical" OR "Plants, Pharmaceutical" OR "Healing Plants" OR "Healing Plant" OR "Plant, Healing" OR "Plants, Healing" ) )

**Web of science-1023**

**TS=** ( "Bronchitis, Chronic" OR "Chronic Bronchitis" ) 20266

**TS=**(“Plants” OR “Plant” OR "Plants, Medicinal" OR “Medicinal Plant” OR “Plant, Medicinal” OR “Medicinal Plants” OR “Medicinal Herbs” OR “Herb, Medicinal” OR “Medicinal Herb” OR “Herbs, Medicinal” OR “Pharmaceutical Plants” OR “Pharmaceutical Plant” OR “Plant, Pharmaceutical” OR “Plants, Pharmaceutical” OR “Healing Plants” OR “Healing Plant” OR “Plant, Healing” OR “Plants, Healing”) 6657835

(TS= ( "Bronchitis, Chronic" OR "Chronic Bronchitis" ))AND( TS=(“Plants” OR “Plant” OR "Plants, Medicinal" OR “Medicinal Plant” OR “Plant, Medicinal” OR “Medicinal Plants” OR “Medicinal Herbs” OR “Herb, Medicinal” OR “Medicinal Herb” OR “Herbs, Medicinal” OR “Pharmaceutical Plants” OR “Pharmaceutical Plant” OR “Plant, Pharmaceutical” OR “Plants, Pharmaceutical” OR “Healing Plants” OR “Healing Plant” OR “Plant, Healing” OR “Plants, Healing”))

**Embase-684**

**Session Results**

| No. | Query Results | Results Date 30 Aug 2024 |
| --- | --- | --- |
| #12. | #4 AND #11 | 684 |
| #11. | #5 OR#6 OR #7 OR #8 OR #9 OR #10 | 1,822,960 |
| #10. | 'plants, medicinal' OR 'plant, medicinal' OR 'medicinal plants' OR 'medicinal herbs' OR 'herb, medicinal' OR 'medicinal herb' OR'herbs,medicinal'OR'pharmaceutical plants'OR'pharmaceuticalplant'OR'plant,pharmaceutical'OR 'plants, pharmaceutical' OR 'healing plants'OR 'healing plant' OR 'plant, healing' OR 'plants, healing' | 78,943 |
| #9. | 'medicinal plant'/exp OR 'medicinal plant' | 345,725 |
| #8. | 'medicinal plant'/exp | 331,026 |
| #7. | 'plants' | 428,449 |
| #6. | 'plant'/exp OR 'plant' | 1,751,985 |
| #5. | 'plant'/exp | 1,292,480 |
| #4. | #1 OR #2 OR #3 | 21,473 |
| #3. | 'bronchitis chronica' OR 'bronchitis, chronic' OR 'chronic bronchus infection' OR 'chronic bronchitis' | 21,426 |
| #2. | 'chronic bronchitis'/exp OR 'chronic bronchitis' | 21,394 |
| #1. | 'chronic bronchitis'/exp | 14,052 |

**CNKI-681**

**P:** chronic bronchitis

**I：**plant OR medicinal plant OR herb OR medicinal herbs

**Wanfang-626 VIP-510**

Title or Keywords :(chronic bronchitis ) and Title or Keywords :(plant OR medicinal plant OR herb OR medicinal herbs)

**SinoMed/CBM-611**

1) "chronic bronchitis"[Common fields: Intelligence] OR "bronchitis, chronic"[Common fields: Intelligence] OR "chronic"[Common fields: Intelligence] AND "bronchitis"[Common fields: Intelligence] OR "bronchitis,"[Common fields: Intelligence] AND "chronic"[Common fields: Intelligence] 15002 2024-08-30 18:41:54.0

2) ((("plant"[Unweighted: Expansion]) OR " medicinal plant"[Unweighted: Expansion]) OR "herb"[Unweighted: Expansion]) OR "t medicinal herbs"[Unweighted: Expansion] 405749 2024-08-30 18:42:46.0

3) (#2) AND (#1) 611
